# Cumulative exposure to remnant cholesterol and the risk of fragility fractures: a longitudinal cohort study

**DOI:** 10.3389/fendo.2023.1251344

**Published:** 2023-11-28

**Authors:** Xiaoli Hou, Nan Zhang, Lu Guo, Yongheng Wang, Mengyi Zheng, Shuohua Chen, Peipei Liu, Mengqin Wang, Jia Li, Shouling Wu, Faming Tian

**Affiliations:** ^1^ School of Public Health, North China University of Science and Technology, Tangshan, China; ^2^ Orthopedics Department, Kailuan General Hospital, Tangshan, China; ^3^ Cardiology Department, Capital Medical University, Beijing, China; ^4^ Cardiology Department, Kailuan General Hospital, Tangshan, China; ^5^ Emergency Department, Jishuitan, The Fourth Clinical Hospital of Peking University, Beijing, China; ^6^ Jitang College, North China University of Science and Technology, Tangshan, China

**Keywords:** remnant cholesterol, cumulative exposure, fragility fractures, bone metabolism, cohort study

## Abstract

**Objective:**

To investigate the association between cumulative remnant cholesterol (cumRC) and the risk of new-onset fragility fractures.

**Methods:**

This study included individuals who participated in the 2006, 2008, and 2010 Kailuan health examinations. Baseline characteristics were compared between groups according to cumRC quartiles. The incidence density was calculated, and the log-rank test was used to compare the cumulative incidence. Cox proportional hazards models were used to estimate the hazard ratio (HR) and 95% confidence interval (CI), and restricted cubic spline was used to examine the possibly non-linear relation between cumRC and the risk of fragility fractures. Additional analyses were performed with stratification by age (≥ or <65 years).

**Results:**

A total of 43,839 individuals were included in this study. During the median follow-up period of 10.97 years, a total of 489 fragility fractures occurred. Multivariable Cox proportional hazards regression model 3 showed that the Q1 and Q4 groups versus the Q2 group were associated with a higher HR of fragility fracture (HR 1.61, 95% CI: 1.23–2.11; HR 1.38, 95% CI: 1.06–1.81), and restricted cubic spline regression analysis showed a non-linear relationship between cumRC level and the risk of fragility fractures (*P*_Overall association_ < 0.001, *P*_Non-linear association_ = 0.001). The association was significant in the age group <65 years but not in the age group ≥65 years. The sensitivity analyses were consistent with the main results.

**Conclusions:**

Both too high and too low cumRC levels were associated with a greater risk of fragility fractures, and this association was more significant in young and middle-aged people.

## Introduction

Fragility fractures are defined as new fractures caused by low trauma (fall from a standing height or less) and mostly affected the hip, spine, distal radius, and proximal humerus (1, 2). Previous data showed that fragility fractures increase the incidence of related complications and even death. Patients with hip fractures have a mortality range of 8.4%–36.0% during the first year following the injury, and nearly 50% are disabled or require long-term home care (25%) (3, 4). High medical costs cause a heavy economic burden for individuals and society. Related risk factors further screened are essential for the prevention of fragility fractures.

In addition to age, gender, bone mineral density, and other indicators in the most widely used fracture risk assessment tool (FRAX) risk prediction system, the relationship between metabolic diseases and related risk factors and fragility fractures is of growing concern. Studies on the association between lipid metabolic disorders and fragility fractures have not shown consistent results. Remnant cholesterol (RC) is a type of cholesterol content that is rich in triglyceride lipoproteins and has been of extensive interest for it is associated with adverse cardiovascular events such as atherosclerosis (5), ischemic heart disease (6), and coronary heart disease (7). A longitudinal cohort study performed in China indicated a superior independent association of increased RC levels with new-onset carotid plaque compared with other conventional lipid parameters (8). Considering a close link between lipid metabolism and bone remodeling, we speculate that the level of RC may be associated with the risk of fragility fractures.

Most previous studies on the effect of lipids on fragility fractures were based on a single lipid level. However, the effect of risk factors on adverse outcome events depends not only on the dose of exposure but also on the duration of exposure. A study found that using multiple blood pressure recordings from patients’ electronic health records showed stronger associations with incident cardiovascular disease than a single blood pressure measurement (9). Therefore, to examine the effect of RC on fragility fractures, the current study was conducted in the Kailuan cohort (registration number: chicTR-TNRC-11001489) to analyze the association between RC and the risk of fragility fractures using cumulative RC (cumRC).

## Materials and methods

### Study population

The present study was based on the Kailuan cohort, which includes all employees of Tangshan Kailuan Group. Individuals undergo biennial questionnaire surveys and physical examinations, which are conducted according to uniform standards in 11 hospitals of Kailuan Group. The large prospective cohort study has collected the population data from seven physical examinations, including epidemiological, physical examination, and serologic detection such as total cholesterol (TC), high-density lipoprotein cholesterol (HDL-C), low-density lipoprotein cholesterol (LDL-C), and triglyceride (TG). Individuals were followed through biennial in-person follow-up surveys, and adverse outcome events including fragility fractures were recorded annually. To observe the effect of cumRC exposure level on fragility fractures, this cohort study included individuals who participated in the 2006, 2008, and 2010 Kailuan health examinations with completed blood lipid indicators, and written informed consent was obtained from all individuals. The individuals with a history of fracture or who experienced pathological fractures, traumatic fractures, and fractures other than hip, chest, and long bones that occurred during the follow-up period were excluded from the study population. This study complied with the principles of the Declaration of Helsinki and was approved by the Ethics Committee of Kailuan Medical Group.

### Measurement of the exposure and covariates

Information on age, gender, physical exercise, smoking, drinking, education, disease history, and medication was obtained through questionnaires. Blood samples were collected after an 8-h fast, and various biochemical parameters including fasting blood glucose (FBG), TC, LDL-C and HDL-C, hemoglobin (Hgb), hypersensitive C-reactive protein (hs-CRP), and creatinine were measured using an automated biochemical analyzer (Hitachi 7600, Tokyo, Japan). TC was measured by the enzymatic colorimetric method, and HDL-C and LDL-C were measured by the direct method. FBG was measured using the hexokinase/glucose‐6‐phosphate‐dehydrogenase method. The estimated filtration rate (eGFR) was calculated with the Chronic Kidney Disease Epidemiology Collaboration creatinine equation, according to creatinine, age, and gender. Seated resting blood pressure was measured using a calibrated mercury sphygmomanometer and stethoscope. Height and weight were measured using the RGZ-120 scale. Body mass index (BMI) was calculated as weight in kilograms divided by height in meters squared. All procedures were carried out strictly by the instructions, and all tests were performed by professional technicians.

The calculated RC was obtained by subtracting LDL-C and HDL-C from the TC measurement (10). The cumRC was calculated as the area under the RC curve from the 2006 to 2010 physical examinations, and the computed formula was as follows: cumRC = (RC_06_ + RC_08_)/2 * time_06–08_ + (RC_08_ + RC_10_)/2 * time_08–10_.

### Relevant definitions and diagnostic criteria

Drinking was defined as consuming ≥100 ml of liquor (alcohol content >50%) per day for more than 1 year. Smoking was defined as smoking one or more cigarettes per day for more than 1 year. Physical exercise was defined as engaging in physical exercise for ≥3 times per week, at least 30 min in duration per time. The educational level was categorized as below high school and high school or above. Hypertension was defined as systolic blood pressure (SBP) ≥140 mmHg and/or diastolic blood pressure (DBP) ≥90 mmHg, or systolic blood pressure <140 mmHg and diastolic blood pressure <90 mmHg, but using antihypertensive drugs or having a history of hypertension. Diabetes was defined as FBG ≥7.0 mmol/L and/or current use of antidiabetic drugs or history of diabetes despite FBG <7.0 mmol/L. Fragility fractures were defined using the codes from the Tenth Revision of the International Classification of Diseases (ICD‐10). Individuals who were hospitalized with an ICD‐10 code about fractures (S22, S32.0, S42.2, S42.3, S42.4, S52, S72, and S82) were considered to have developed fragility fractures.

### Assessment of follow-up and outcomes

The cohort follow-up was from the end of the 2010 physical examination until the fragility fracture date, the death date, or the last follow-up date (31 December 2021). The primary outcome was the first occurrence of fragility fractures. During the follow-up period, the fragility fracture events and deaths were tracked through the medical records system of the Kailuan Medical Insurance system, Kailuan General Hospital, and its affiliated mining hospitals. The outcome information was further confirmed by trained investigators from the hospitals where individuals were treated and diagnosed.

### Statistical analysis

Normally distributed continuous data were presented as mean ± standard deviations (SD), and a comparison between groups was presented using a variance. Skewed distributions were given as median (interquartile range) and compared using the non-parametric Wilcoxon rank sum test. Categorical data were presented as percentages and compared between groups by the chi-square test. Baseline characteristics were compared between groups according to cumRC quartiles. The incidence density of fragility fractures was calculated as the number of incident cases relative to the number of person-years contributed by the study individuals (1,000/person-year). Kaplan–Meier curves were plotted, and the log-rank test was used to compare the cumulative incidence of fragility fractures in each group. The proportional hazards assumption for all Cox proportional hazards regression models was verified by Schoenfeld residuals. Cox proportional hazards models were used to estimate the hazard ratio (HR) and 95% confidence interval (CI) of fragility fractures. Multivariate models adjusted the covariates: model 1 was adjusted for age, gender, education, smoking, drinking, physical activity, and BMI; model 2 was further adjusted for hs-CRP, eGFR, Hgb, history of diabetes, history of hypertension, history of cardiovascular disease (CVD), history of cancer, and history of atrial fibrillation (AF). Model 3 was adjusted for lipid-lowering therapy based on model 2. Model 4 was adjusted for the RC level measured at the 2006 (RC_2006_) physical examination based on model 3. A restricted cubic spline (RCS) was used to examine the possibly non-linear relation between cumRC and fragility fractures. Additional analyses were performed with stratification by age (≥ or <65 years). Sensitivity analyses were performed by removing new-onset fragility fractures within 1 year of follow-up and removing individuals with a history of CVD, cancer, and AF or taking lipid-lowering medications. An analysis adjusting for death as a competing risk provided a sensitivity analysis. Finally, the time-weighted average RC (twaRC) was calculated by the formula—cumRC/time_06–10_—and was taken into the Cox proportional hazards regression models. In the sensitivity analysis, TC, HDL-C, LDL-C, TG, SBP, and DBP were further adjusted. Data analysis was performed using SAS9.4 and R version 4.2.1. *P* <0.05 (two-sided test) was regarded as statistically significant.

## Results

### General characteristics at baseline

**Figure 1:**
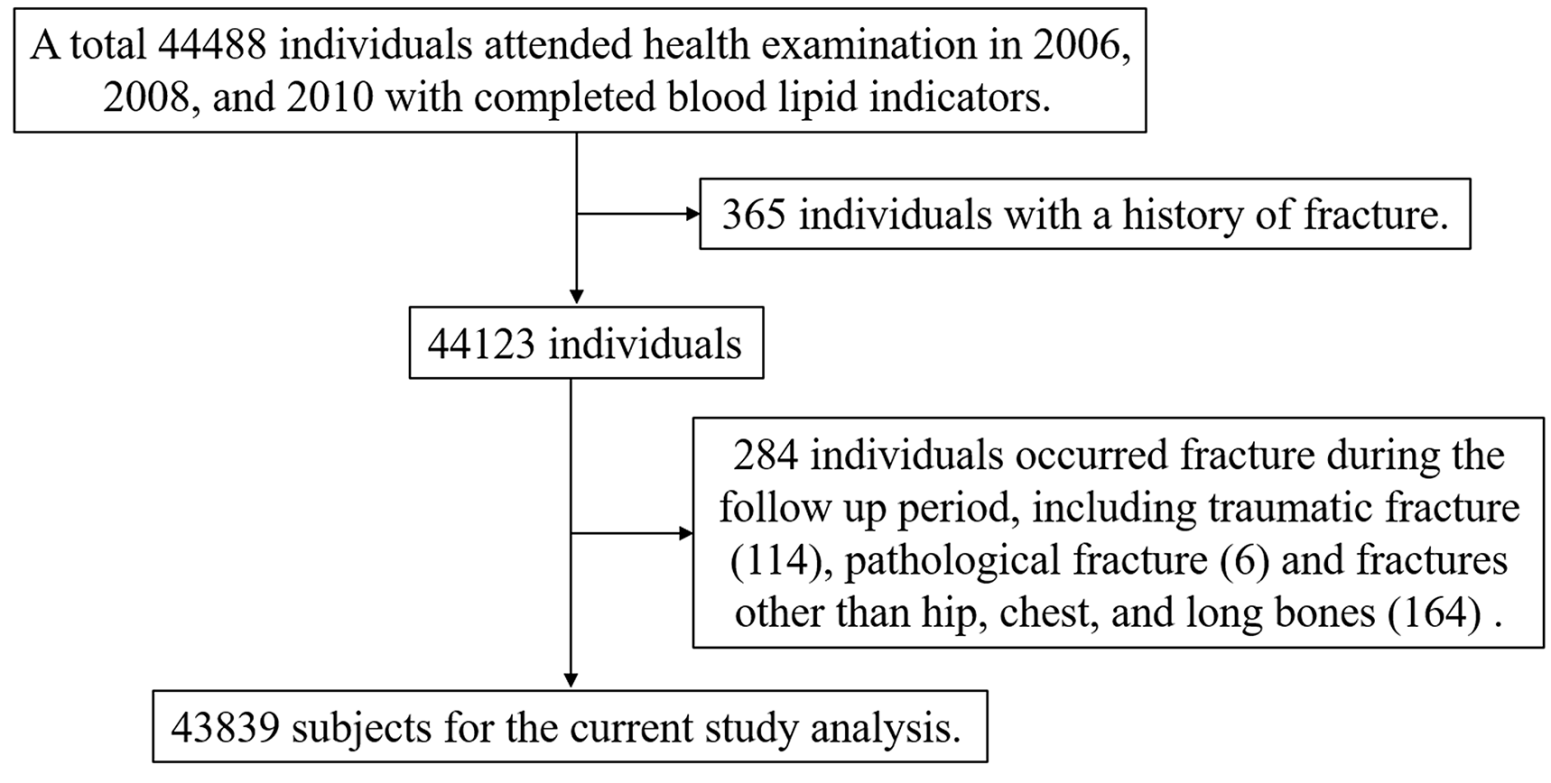
Flowchart of the current study population.

A total of 44,488 individuals participated in the 2006, 2008, and 2010 annual physical examinations, and 43,839 fulfilled the inclusion/exclusion criteria and were finally included in the analysis (**Figure 1**), consisting of 34,292 men (78.22%) and 9,547 women (21.78%). The mean (SD) age of the study population at baseline (2010) was 53.58 ± 11.96 years old. According to the cumRC quartile, the individuals were divided into quartile 1 (Q1), Q2, Q3, and Q4 groups, and the baseline characteristics were compared among the groups. The results showed that individuals in the higher cumRC group had higher levels of BMI, FBG, SBP, DBP, TG, TC, twaRC, and RC_06_; a higher proportion of lipid-lowering therapy; and a higher incidence of diabetes, hypertension, and CVD (*P* < 0.001) (**Table 1**).

**Table 1:** Baseline characteristics according to baseline.

Characteristics	Overall	Q1	Q2	Q3	Q4	*P*-value
Participants, *n* (%)	43,839	10,960	10,959	10,961	10,959	
Age (years), mean ± SD	53.58 ± 11.96	51.81 ± 12.65	52.69 ± 12.43	53.97 ± 11.56	55.86 ± 10.71	<0.001
Males, *n* (%)	34,292 (78.22)	8,471 (77.29)	8,744 (79.79)	8,571 (78.20)	8,506 (77.62)	<0.001
Smoker, *n* (%)	16,775 (38.27)	3,516 (32.08)	4,223 (38.53)	4,565 (41.65)	4,471 (40.80)	<0.001
Drinker, *n* (%)	15,276 (34.85)	3,282 (29.95)	3,947 (36.02)	4,110 (37.50)	3,937 (35.92)	<0.001
Physical exercise, *n* (%)	6,507 (14.84)	1,481 (13.51)	1,649 (15.05)	1,708 (15.58)	1,669 (15.23)	<0.001
High school or above educational level, *n* (%)	11,766 (26.84)	3,144 (28.69)	3,305 (30.16)	2,825 (25.77)	2,492 (22.74)	<0.001
BMI (kg/m^2^), mean ± SD	25.20 ± 3.37	24.54 ± 3.30	25.21 ± 3.41	25.36 ± 3.36	25.69 ± 3.29	<0.001
Hgb (g/L), mean ± SD	147.61 ± 18.95	147.39 ± 17.67	147.95 ± 18.86	147.61 ± 18.99	147.51 ± 20.18	<0.001
FBG (mmol/L), mean ± SD	5.67 ± 1.52	5.45 ± 1.260	5.62 ± 1.44	5.72 ± 1.54	5.90 ± 1.77	<0.001
hs-CRP (mg/L), median (IQR)	1.20 (0.60–2.80)	1.20 (0.59–2.70)	1.12 (0.50–2.65)	1.20 (0.60–2.80)	1.30 (0.60–3.04)	<0.001
SBP (mmHg), mean ± SD	131.35 ± 19.56	129.24 ± 19.85	130.40 ± 19.62	131.72 ± 19.34	134.02 ± 19.11	<0.001
DBP (mmHg), mean ± SD	84.36 ± 10.93	82.85 ± 11.00	84.02 ± 10.85	84.94 ± 10.96	85.62 ± 10.69	<0.001
eGFR, mean ± SD	90.15 ± 19.120	86.84 ± 21.11	90.44 ± 19.78	92.26 ± 17.76	91.06 ± 17.13	<0.001
TG (mmol/L), mean ± SD	1.66 ± 1.370	1.08 ± 0.75	1.49 ± 0.94	1.75 ± 1.20	2.32 ± 1.95	<0.001
HDL-C (mmol/L), mean ± SD	1.53 ± 0.43	1.59 ± 0.41	1.52 ± 0.42	1.50 ± 0.43	1.50 ± 0.44	<0.001
LDL-C (mmol/L), mean ± SD	2.61 ± 0.80	2.59 ± 0.71	2.62 ± 0.73	2.62 ± 0.81	2.59 ± 0.94	<0.001
TC (mmol/L), mean ± SD	5.07 ± 0.96	4.67 ± 0.82	4.90 ± 0.86	5.08 ± 0.87	5.64 ± 1.01	<0.001
RC_06_ (mmol/L), mean ± SD	1.29 ± 0.84	0.57 ± 0.33	1.04 ± 0.51	1.44 ± 0.61	2.10 ± 0.90	<0.001
cumRC (mmol/L × year), mean ± SD	4.29 ± 2.11	1.89 ± 0.51	3.33 ± 0.38	4.74 ± 0.45	7.20 ± 1.34	<0.001
twaRC (mmol/L), mean ± SD	1.06 ± 0.52	0.48 ± 0.13	0.83 ± 0.12	1.18 ± 0.16	1.75 ± 0.36	<0.001
Lipid-lowering therapy, *n* (%)	4,811 (10.97)	797 (7.27)	1,008 (9.20)	1,209 (11.03)	1,797 (16.40)	<0.001
Hypertension, *n* (%)	20,863 (47.59)	4,354 (39.73)	5,028 (45.88)	5,434 (49.58)	6,047 (55.18)	<0.001
Diabetes, *n* (%)	6,901 (15.74)0	1,293 (11.80)	1,623 (14.81)	1,793 (16.36)	2,192 (20.00)	<0.001
CVD, *n* (%)	1,729 (3.94)	300 (2.74)	396 (3.61)	481 (4.39)	552 (5.04)	<0.001
Cancer, *n* (%)	437 (1.00)	106 (0.97)	99 (0.90)	123 (1.12)	109 (0.99)	0.4214
AF, *n* (%)	216 (0.49)	48 (0.44)	47 (0.43)	55 (0.50)	66 (0.60)	0.2326

Values are n (%) or mean (standard deviation) or median (interquartile range).

SD, standard deviation; IQR, interquartile range; BMI, body mass index; Hgb, hemoglobin; hs-CRP, hypersensitive C-reactive protein; FPG, fasting plasma glucose; eGFR, estimated glomerular filtration rate; SBP, systolic blood pressure, DBP, diastolic blood pressure; TG, triglyceride; HDL, high-density lipoprotein; LDL, low-density lipoprotein; TC, total cholesterol; cumRC, cumulative remnant cholesterol; twaRC, time-weighted average remnant cholesterol; RC_06_, remnant cholesterol was measured by physical examination in 2006; CVD, cardiovascular disease; AF, atrial fibrillation.

### The incidence of fragility fractures

**Figure 2:**
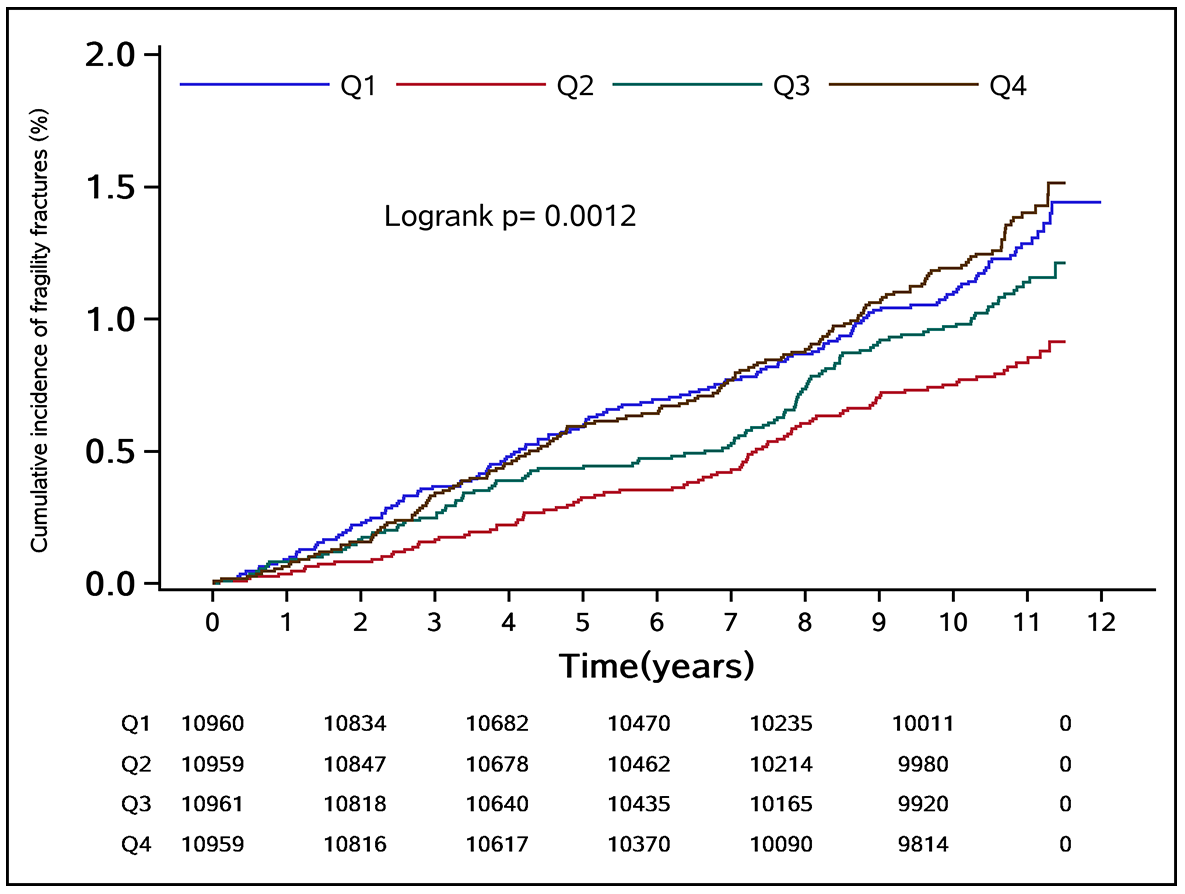
Kaplan–Meier curves for the cumulative incidence of fragility fractures.

During the median follow-up period of 10.97 years, a total of 489 fragility fractures occurred in the study population, with an incidence density of 1.06 (95% CI: 0.98–1.16) per 1,000 person-years. Of these, 342 were men with an incidence density of 0.96 (95% CI: 0.86–1.06) per 1,000 person-years, and 147 were women with an incidence density of 1.45 (95% CI: 1.23–1.70) per 1,000 person-years. The cumulative incidence of fragility fractures in groups Q1–Q4 was 1.11%, 0.76%, 0.98%, and 1.20%, respectively. The difference in cumulative incidence of fragility fractures among the groups was statistically significant (log-rank test *P* = 0.0012) (**Figure 2**).

### The cumRC and fragility fracture risk

**Figure 3:**
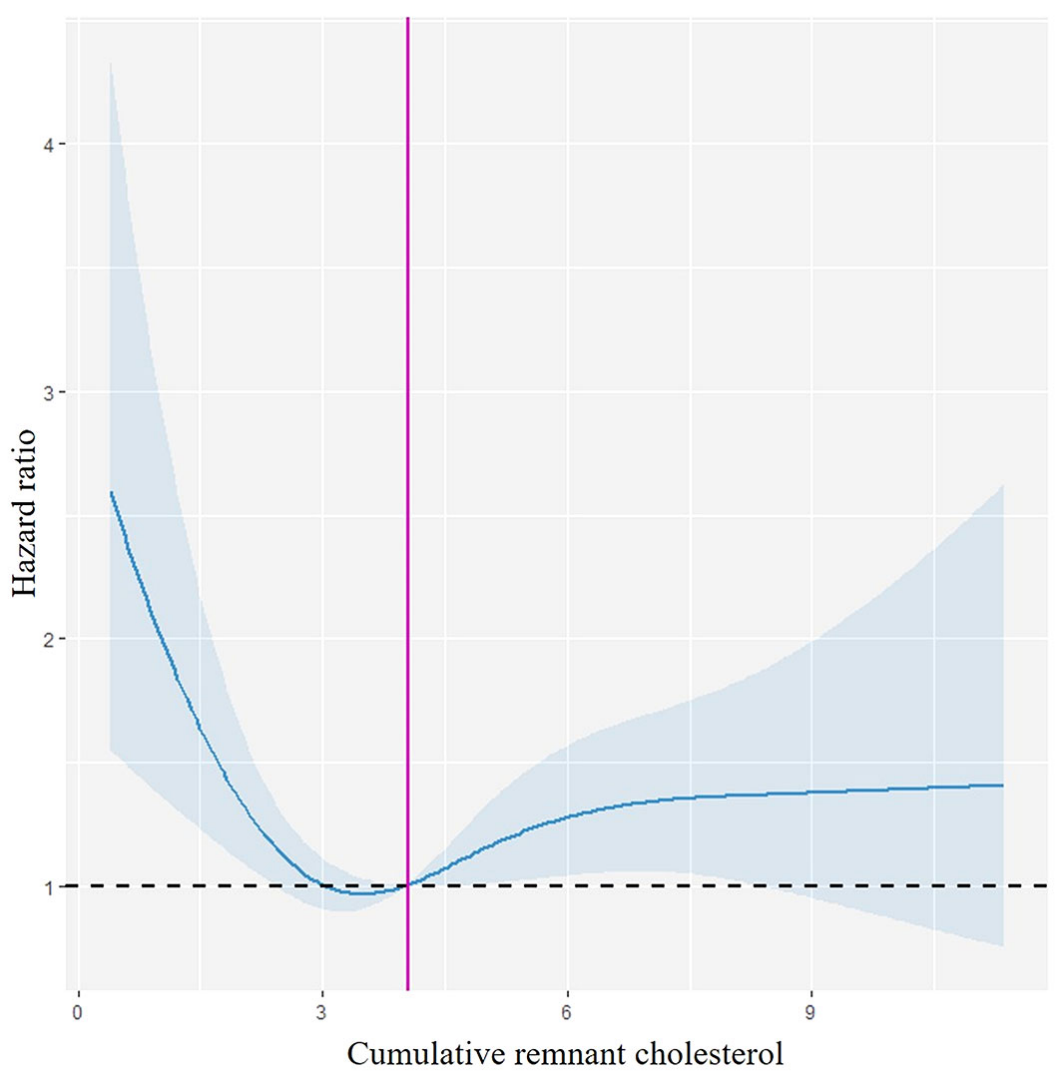
Restricted cubic spline curve. Spline curves demonstrating the relationship between the cumRC level and the risk of fragility fracture events, with 95% CI depicted in light blue. This model is adjusted for age, gender, education, drinking, physical exercise, hs-CRP, eGFR, Hgb, CVD, AF, cancer, diabetes, hypertension, lipid-lowering therapy, and RC_06_.

Following adjusting for potential confounding factors, Cox proportional hazards regression analysis showed that the Q1 and Q4 groups versus the Q2 group were associated with a higher HR of fragility fractures (HR 1.61, 95% CI: 1.23–2.11; HR 1.38, 95% CI: 1.06–1.81) in model 3 (**Table 2**). Multivariable adjusted RCS regression analysis showed a non-linear relationship between cumRC level and the risk of fragility fractures (*P*_Overall association_ < 0.001, *P*_Non-linear association_ = 0.001) (**Figure 3**).

**Table 2:** Association of cumRC exposure with fragility fracture risk (*n* = 43,839).

	Q1	Q2	Q3	Q4
Cases, *n* (%)	138 (1.26)	89 (0.81)	118 (1.08)	144 (1.31)
Incidence rate, per 1,000 person-years	1.20 (1.01–1.41)	0.77 (0.63–0.95)	1.03 (0.86–1.23)	1.27 (1.08–1.49)
Person-years	115,413.53	115,199.50	114,915.64	113,685.90
Model 1, HR (95% CI)	1.62 (1.24-2.12)	Ref	1.23 (0.93–1.62)	1.40 (1.07–1.82)
Model 2, HR (95% CI)	1.61 (1.23-2.11)	Ref	1.23 (0.94–1.62)	1.39 (1.06–1.81)
Model 3, HR (95% CI)	1.61 (1.23-2.11)	Ref	1.23 (0.93–1.62)	1.38 (1.06–1.81)
Model 4, HR (95% CI)	1.53 (1.16-2.01)	Ref	1.29 (0.97–1.71)	1.57 (1.15–2.13)

Model 1: adjusted for age, gender, education, drinking, smoking, physical exercise, and BMI.

Model 2: model 1 + hs-CRP, eGFR, Hgb, CVD, AF, cancer, diabetes, and hypertension.

Model 3: model 2 + lipid-lowering therapy.

Model 4: model 3 + RC_06_.

Q1: cumRC <2.67, Q2: 2.67 ≤ cumRC < 4.01, Q3: 4.01 ≤ cumRC < 5.58, Q4: ≥5.58.

### Subgroup analysis

The interaction effect test between the group and all covariates showed that age by group interaction was statistically significant in a model adjusted for other covariates (*P* = 0.017). The cohort was stratified for further analysis by age greater or less than 65. Multivariate adjusted Cox proportional hazards regression model 3 analysis showed that in the age group <65 years, the Q1 and Q4 groups versus the Q2 group were associated with a higher HR of fragility fractures (HR 1.99, 95% CI: 1.41–2.80; HR 1.94, 95% CI: 1.37–2.74). However, in the age group ≥65 years, the HRs of Q1 and Q4 were 0.97 (95% CI: 0.61–1.52) and 0.89 (95% CI: 0.58–1.37) compared with the Q2 group, respectively (**Table 3**).

**Table 3:** Subgroup analysis by age.

		Q1	Q2	Q3	Q4
Age <65 *n* = 36,683	Cases, *n* (%)	101 (1.09)	49 (0.53)	83 (0.90)	98 (1.09)
	Incidence rate, per 1,000 person-years	1.01 (0.83–1.23)	0.50 (0.37–0.66)	0.84 (0.68–1.04)	1.03 (0.84–1.25)
	Person-years	99,866.52	98,998.18	98,883.98	95,407.65
	Model 1, HR (95% CI)	2.04 (1.45–2.87)	Ref	1.64 (1.16–2.34)	1.99 (1.41–2.81)
	Model 2, HR (95% CI)	1.98 (1.41–2.80)	Ref	1.66 (1.16–2.36)	1.94 (1.37–2.74)
	Model 3, HR (95% CI)	1.99 (1.41–2.80)	Ref	1.66 (1.16–2.36)	1.94 (1.37–2.74)
	Model 4, HR (95% CI)	1.89 (1.33–2.69)	Ref	1.73 (1.21–2.48)	2.16 (1.47–3.19)
Age ≥65 *n* = 7,156	Cases, *n* (%)	37 (2.20)	40 (2.28)	35 (1.99)	46 (2.34)
	Incidence rate, per 1,000 person-years	2.38 (1.72–3.28)	2.47 (1.81–3.37)	2.18 (1.57–3.04)	2.52 (1.89–3.36)
	Person-years	15,547.00	16,201.33	16,031.66	18,278.25
	Model 1, HR (95% CI)	0.97 (0.61–1.52)	Ref	0.82 (0.52–1.29)	0.92 (0.60–1.41)
	Model 2, HR (95% CI)	0.97 (0.61–1.53)	Ref	0.80 (0.51–1.27)	0.90 (0.58–1.38)
	Model 3, HR (95% CI)	0.97 (0.61–1.52)	Ref	0.80 (0.51–1.26)	0.89 (0.58–1.37)
	Model 4, HR (95% CI)	0.92 (0.57–1.47)	Ref	0.83 (0.52–1.33)	1.01 (0.60–1.70)

Model 1: adjusted for gender, education, drinking, physical exercise, and BMI.

Model 2: model 1 + hs-CRP, eGFR, Hgb, CVD, AF, cancer, diabetes, and hypertension.

Model 3: model 2 + lipid-lowering therapy.

Model 4: model 3 + RC_06_.

### Sensitivity analysis

The above analysis was repeated using a multivariate adjusted Cox proportional hazards regression model. To reduce the possibility of reverse causation, 30 individuals developing fragility fractures less than 1 year after baseline were excluded, and this did not affect the results (**Table S1**). To rule out the potential effects of CVD, AF, and cancer, 2,306 individuals with these conditions at baseline were excluded, but this did not change the results (**Table S2**). To exclude the effect of lipid-lowering therapy, 4,811 individuals taking lipid-lowering drugs were excluded, and the results did not change significantly (**Table S3**). The competing risk regression model in which we considered dying without fragility fractures as the competing event led to consistent results (**Table S4**). Additionally, twaRC was included as an independent variable in the Cox proportional hazards regression models, and the results were consistent with the primary analyses, with *P*_Overall association_ < 0.001 and *P*_Non-linear association_ < 0.001 (**Table S5**, **Figure S1**). The sensitivity analyses with further adjustments for TC, HDL-C, LDL-C, TG, SBP, and DBP yielded similar effect estimates (**Table S6**).

## Discussion

This study provides the report of a U-shaped relationship between the cumRC level and the risk of developing fragility fractures. Both too high and too low cumRC levels were associated with a greater risk of fragility fractures, with the effect being more significant in young and middle-aged people (age <65 years). To the best of our knowledge, this is the first study to investigate the association between cumRC exposure and fragility fractures, and our findings further confirm that dyslipidemia increases the risk of fragility fractures.

Following adjustment for potential confounders, compared with the Q2 group with the lowest event rate, the risk of new-onset fragility fractures increased by 61% and 38% in the Q1 and Q4 groups, respectively, and maybe the result was affected by the single RC measurement but remained independent of it. Further analysis using RCS regression showed a non-linear relationship between cumRC exposure and the risk of fragility fractures. While no similar studies have been conducted to date, the effect of traditional lipid indicators on fragility fractures has been reported. A meta-analysis showed that each 50 mg/dl increase in TC was associated with a 15% increase in fracture risk (11). In contrast, a prospective cohort study in Austria revealed that women with higher TC had a reduced risk of hip fracture (12). A nested case–control study from Denmark showed that lower LDL-C levels were associated with an increased risk of fracture, as compared with LDL-C measurements between 3.039 mmol/L and 5.959 mmol/L. LDL-C measurements equal to or higher than 3 mmol/L were protective against fragility fractures among patients with diabetes (13). Different from the previous observation of a single blood lipid level, the present study evaluated the risk of fragility fractures using the cumulative exposure value calculated from three RC measurements over 6 years. The results reflected the association between long-term RC burden and the risk of new-onset fragility fractures, and the sensitivity analyses were supportive of the main analysis.

However, when further stratified by age (<65 years and ≥65 years), the association between cumRC and fragility fractures was significant in the age group <65 years, but not in the age group ≥65 years. According to research, the primary risk factors for osteoporosis in individuals younger than the age of 55 are modifiable, while non-modifiable risk factors such as reduced hormone levels, chronic pain, and sleep disorders become crucial in individuals older than 55 (14). It is suggested that lowering exposure to risk factors may be more beneficial for the primary prevention of osteoporosis in younger populations. In older individuals, the risk of cardiovascular disease, chronic inflammation, glucose, and lipid disorders increases. Although a single disease may not significantly affect bone density, the simultaneous occurrence of multiple diseases is associated with the accumulation of physiological system imbalances and a decrease in bone strength. Moreover, the decline in muscle mass caused by a lack of physical activity in the elderly may increase the risk of falls and decrease bone density. Therefore, the effect of cumRC exposure on fragility fractures may be overlaid by aging in the elderly population. Although the incidence of fracture is lower in younger populations, the risk of secondary fractures may be increased after the first fracture. Our research results may provide insights into strategies for preventing fragility fractures in younger populations. Statins are the commonly used lipid-lowering agents, but the effects of statins on osteoblast differentiation and bone formation remain controversial (15). Although the HR value did not change appreciably in either the Q1 or Q4 groups compared with model 2 after further adjusting for lipid-lowering therapy in model 3, in a sensitivity analysis excluding people taking statins, the HR value decreased in the Q1 group, suggesting that statin lipid-lowering drugs may increase the risk of fragility fractures caused by low RC. Therefore, not only the lipid-lowering effect of statins but also the increased risk of fragility fractures should be considered when they are prescribed for those with lower levels of RC.

Previous studies showed U-shaped or reverse U-shaped relationships between some adverse health events and lipid levels (16, 17), similar to the present study between cumRC and fragility fracture risk, suggesting a possible dual effect of lipid metabolism on bone metabolism. On the one hand, it has been found that increased lipids accumulate beneath the vascular intima and perivascular space in the bones, and the inflammatory bioactive lipids induce bone loss; particularly, oxidized LDL-C plays a significant role in bone loss. Lipid oxidation products can promote arterial calcification by activating osteoblasts but inhibit bone formation in bone tissue (18). Lower Runx2 expression and higher TRAP expression were found in both diet-induced and genetic hyperlipidemia mice, indicating decreased osteoblastic functions and increased osteoclastic functions in these mice (19). Moreover, hyperlipidemia may also induce secondary hyperparathyroidism, further impairing bone regeneration and compromising mechanical strength (20). Finally, the accumulation of fat in the femoral head has been observed to elevate the pressure within the bone marrow microcirculation, resulting in a reduction in bone vascularization. This diminished blood supply causes ischemia and hypoxia in the affected area, impairing the blood supply to the bone. All these factors may contribute to an increased risk of fractures (18). On the other hand, it has been proven that exogenous cholesterol inhibits osteoblast differentiation, while endogenous cholesterol may promote osteogenic differentiation of bone marrow mesenchymal stem cells by activating the Hedgehog signaling pathway (21). Cholesterol may inhibit autophagy during osteoclast differentiation by activating the phosphatidylinositol 3-kinase/AKT/mammalian target of rapamycin signaling pathway and play a role in bone resorption and formation (22). These studies suggest that the effect of cholesterol on osteogenesis is more complex than either “bad” or “good.” Noteworthy, in the present study, the HR for fragility fractures was higher in the Q1 than in the Q4 group. It has been found that cholesterol loading promotes osteogenic differentiation of mesenchymal stem cells and mainly depends on the effect of cholesterol esters, which can promote the formation of mineralized nodules by increasing bone morphogenetic protein 2 and runt-related transcription factor 2 expression (23). The RC particle is larger and carries more cholesterol than other types of cholesterol, and the negative effect of cumRC decrease on osteogenesis may be more obvious, but the specific mechanism remains to be studied.

The main strengths of the present study are the large number of samples and the long follow-up period. Compared with previous studies on the association between single lipid exposure and the risk of fragility fractures, this study used repeated measurement data, which provides a theoretical basis for the application of cumulative exposure. However, there are some limitations. Firstly, the lack of bone mineral density data limits the further exploration of the problems and potential reasons, such as whether the association between cumRC and the risk of fragility fractures is affected by bone mineral density. Second, the large sample was recruited from the data from north China, and whether these results apply to other countries or populations remains unknown. Third, information about potential risk factors such as parental fragility fracture history was not obtained, so there may be residual confounding in the study. Fourth, the data on the level of vitamin D hormone drugs, the usage of steroids, and other bone metabolic markers were not obtained from the participants.

## Conclusion

In conclusion, both too high and too low cumRC exposure increased the risk of fragility fractures, and the effect was more significant in young and middle-aged people independent of a single RC measurement. Therefore, much more attention should be devoted to the harm caused by cumRC exposure for both clinicians and individuals and control the RC level in the ideal range as much as possible to reduce the risk of fragility fractures and improve the quality of life.

## Data availability statement

The data that support the findings of this study are available on request from the corresponding author.

## Ethics statement

The studies involving humans were approved by the Ethics Committee of Kailuan Medical Group. The studies were conducted in accordance with the local legislation and institutional requirements. The participants provided their written informed consent to participate in this study.

## Author contributions

FT and SW designed the study and edited the manuscript. XH developed the manuscript draft and statistical analyses. NZ and LG collected the data and conducted the investigation. YW, MZ, and SC conducted the investigation and methodology. PL, MW, and JL took responsibility for the integrity of the data. All authors contributed to the article and approved the submitted version.
