# Supplementary material for: Cumulative exposure to remnant cholesterol and the risk of fragility fractures: a longitudinal cohort study
Source: Front Endocrinol (Lausanne). 2023 Nov 28;14:1251344. doi: 10.3389/fendo.2023.1251344 (PMC10713996; doi:10.3389/fendo.2023.1251344)
Supplement: Supplementary file 1 [file Table_1.docx]

**Table S1** Sensitivity analysis by excluding outcomes occurred within the first follow-up years (n=43,809)

|  | Q1 | Q2 | Q3 | Q4 |
| --- | --- | --- | --- | --- |
| Cases, n (%) | 128(1.17) | 85(0.78) | 109(1.00) | 137(1.25) |
| Incidence rate,  per 1000 person-years | 1.11(0.93-1.32) | 0.74(0.60-0.91) | 0.95(0.79-1.14) | 1.21(1.02-1.42) |
| Person-years | 115408.32 | 115197.56 | 114910.89 | 113682.26 |
| Model 1 HR (95%CI) | 1.57(1.19-2.07) | ref | 1.19(0.90-1.58) | 1.39(1.06-1.83) |
| Model 2 HR (95%CI) | 1.56(1.18-2.06) | ref | 1.19(0.90-1.58) | 1.38(1.05-1.82) |
| Model 3 HR (95%CI) | 1.56(1.18-2.06) | ref | 1.19(0.90-1.58) | 1.38(1.05-1.81) |
| Model 4 HR (95%CI) | 1.47(1.10-1.95) | ref | 1.25(0.94-1.67) | 1.59(1.16-2.18) |

Model 1: adjusted for age, gender, education, drinking, physical exercise, and BMI.

Model 2: Model 1+hs-CRP, eGFR, Hgb, CVD, AF, cancer, diabetes and hypertension.

Model 3: Model 2+lipid-lowering therapy.

Model 4: Model 3+RC_06_.

**Table S2** Sensitivity analysis by excluding participants with the history of CVD, AF, and cancer (n=41,533)

|  | Q1 | Q2 | Q3 | Q4 |
| --- | --- | --- | --- | --- |
| Cases, n (%) | 129(1.23) | 79(0.76) | 108(1.05) | 130(1.27) |
| Incidence rate,  per 1000 person-years | 1.16(0.97-1.38) | 0.72(0.57-0.89) | 0.99(0.82-1.20) | 1.21(1.02-1.44) |
| Person-years | 111443.57 | 110322.13 | 109065.92 | 107219.29 |
| Model 1 HR (95%CI) | 1.67(1.26-2.22) | ref | 1.29(0.96-1.72) | 1.44(1.09-1.91) |
| Model 2 HR (95%CI) | 1.65(1.24-2.19) | ref | 1.29(0.97-1.73) | 1.44(1.08-1.91) |
| Model 3 HR (95%CI) | 1.65(1.25-2.19) | ref | 1.29(0.96-1.73) | 1.43(1.08-1.90) |
| Model 4 HR (95%CI) | 1.56(1.17-2.10) | ref | 1.35(1.01-1.82) | 1.62(1.17-2.24) |

Model 1: adjusted for age, gender, education, drinking, physical exercise, and BMI.

Model 2: Model 1+hs-CRP, eGFR, Hgb, diabetes and hypertension.

Model 3: Model 2+lipid-lowering therapy.

Model 4: Model 3+RC_06_.

**Table S3** Sensitivity analysis by excluding participants with lipid-lowering drugs (n=39,028)

|  | Q1 | Q2 | Q3 | Q4 |
| --- | --- | --- | --- | --- |
| Cases, n (%) | 122(1.20) | 82(0.82) | 94(0.96) | 117(1.28) |
| Incidence rate,  per 1000 person-years | 1.14(0.95-1.36) | 0.78(0.63-0.97) | 0.92(0.75-1.13) | 1.23(1.03-1.48) |
| Person-years | 107092.88 | 104639.97 | 102215.97 | 95000.63 |
| Model 1 HR (95%CI) | 1.51(1.14-2.00) | ref | 1.10(0.82-1.48) | 1.36(1.03-1.81) |
| Model 2 HR (95%CI) | 1.48(1.11-1.96) | ref | 1.11(0.82-1.49) | 1.38(1.04-1.83) |
| Model 3 HR (95%CI) | 1.38(1.03-1.84) | ref | 1.18(0.87-1.60) | 1.62(1.17-2.25) |

Model 1: adjusted for age, gender, education, drinking, physical exercise, and BMI.

Model 2: Model 1+hs-CRP, eGFR, Hgb, CVD, AF, cancer, diabetes and hypertension.

Model 3: Model 2+RC_06_.

**Table S4** Sensitivity analysis by using competing risk mode (n=43,839)

|  | Q1 | Q2 | Q3 | Q4 |
| --- | --- | --- | --- | --- |
| Model 1 HR (95%CI) | 1.63(1.24-2.12) | ref | 1.24(0.94-1.63) | 1.40(1.07-1.83) |
| Model 2 HR (95%CI) | 1.62(1.23-2.12) | ref | 1.24(0.94-1.64) | 1.40(1.07-1.83) |
| Model 3 HR (95%CI) | 1.62(1.23-2.12) | ref | 1.24(0.94-1.63) | 1.39(1.06-1.82) |
| Model 4 HR (95%CI) | 1.54(1.17-2.03) | ref | 1.29(0.98-1.71) | 1.56(1.14-2.15) |

Model 1: adjusted for age, gender, education, drinking, physical exercise, and BMI.

Model 2: Model 1+hs-CRP, eGFR, Hgb, CVD, AF cancer, diabetes and hypertension.

Model 3: Model 2+lipid-lowering therapy.

Model 4: Model 3+RC_06_.

**Table S5** Association of twaRC level with fragility fractures risk (n=43,839)

|  | Q1 | Q2 | Q3 | Q4 |
| --- | --- | --- | --- | --- |
| Cases, n (%) | 143(1.30) | 96(0.88) | 109(0.99) | 141(1.29) |
| Incidence rate,  per 1000 person-years | 1.25(1.06-1.47) | 0.84(0.68-1.02) | 0.95(0.79-1.14) | 1.23(1.04-1.45) |
| Person-years | 114318.49 | 114946.79 | 114892.56 | 115056.73 |
| Model 1 HR (95%CI) | 1.54(1.19-2.00) | ref | 1.08(0.82-1.42) | 1.35(1.04-1.75) |
| Model 2 HR (95%CI) | 1.53(1.18-1.99) | ref | 1.08(0.82-1.42) | 1.34(1.03-1.75) |
| Model 3 HR (95%CI) | 1.53(1.18-1.99) | ref | 1.08(0.82-1.42) | 1.34(1.03-1.74) |
| Model 4 HR (95%CI) | 1.45(1.11-1.90) | ref | 1.12(0.85-1.49) | 1.52(1.12-2.05) |

Model 1: adjusted for age, gender, education, drinking, physical exercise, and BMI.

Model 2: Model 1+hs-CRP, eGFR, Hgb, CVD, AF, cancer, diabetes and hypertension.

Model 3: Model 2+lipid-lowering therapy.

Model 4: Model 3+RC_06_.

Q1: twaRC<0.66, Q2: 0.66≤twaRC<0.99, Q3: 0.99≤twaRC<1.38, Q4: twaRC≥1.38

**Table S6** Sensitivity analysis with further adjustments for other covariates

|  | Q1 | Q2 | Q3 | Q4 |
| --- | --- | --- | --- | --- |
| Model 1 HR (95%CI) | 1.47(1.24-2.12) | ref | 1.32(0.99-1.75) | 1.71(1.24-2.36) |
| Model 2 HR (95%CI) | 1.47(1.11-1.95) | ref | 1.31(0.98-1.73) | 1.70(1.23-2.34) |

Model 1: adjusted for age, gender, education, drinking, smoking, physical exercise, BMI, hs-CRP, eGFR, Hgb, CVD, AF, cancer, diabetes and hypertension, lipid-lowering therapy, RC_06_, TC, HDL-C, LDL-C and TG.

Model 2: adjusted for Model 1+SBP and DBP.

**Fig.S1**. Restricted cubic spline curve. Spline curves demonstrate the relationship between twaRC level and risk of fragility fracture events, with 95% CI depicted in light blue. This model is adjusted for age, gender, education, drinking, physical exercise, hs-CRP, eGFR, Hgb, CVD, AF, cancer, diabetes, hypertension, lipid-lowering therapy, and RC_06_.
