# Supplementary figures and images for: Cumulative exposure to remnant cholesterol and the risk of fragility fractures: a longitudinal cohort study
Source: Front Endocrinol (Lausanne). 2023 Nov 28;14:1251344. doi: 10.3389/fendo.2023.1251344 (PMC10713996; doi:10.3389/fendo.2023.1251344)

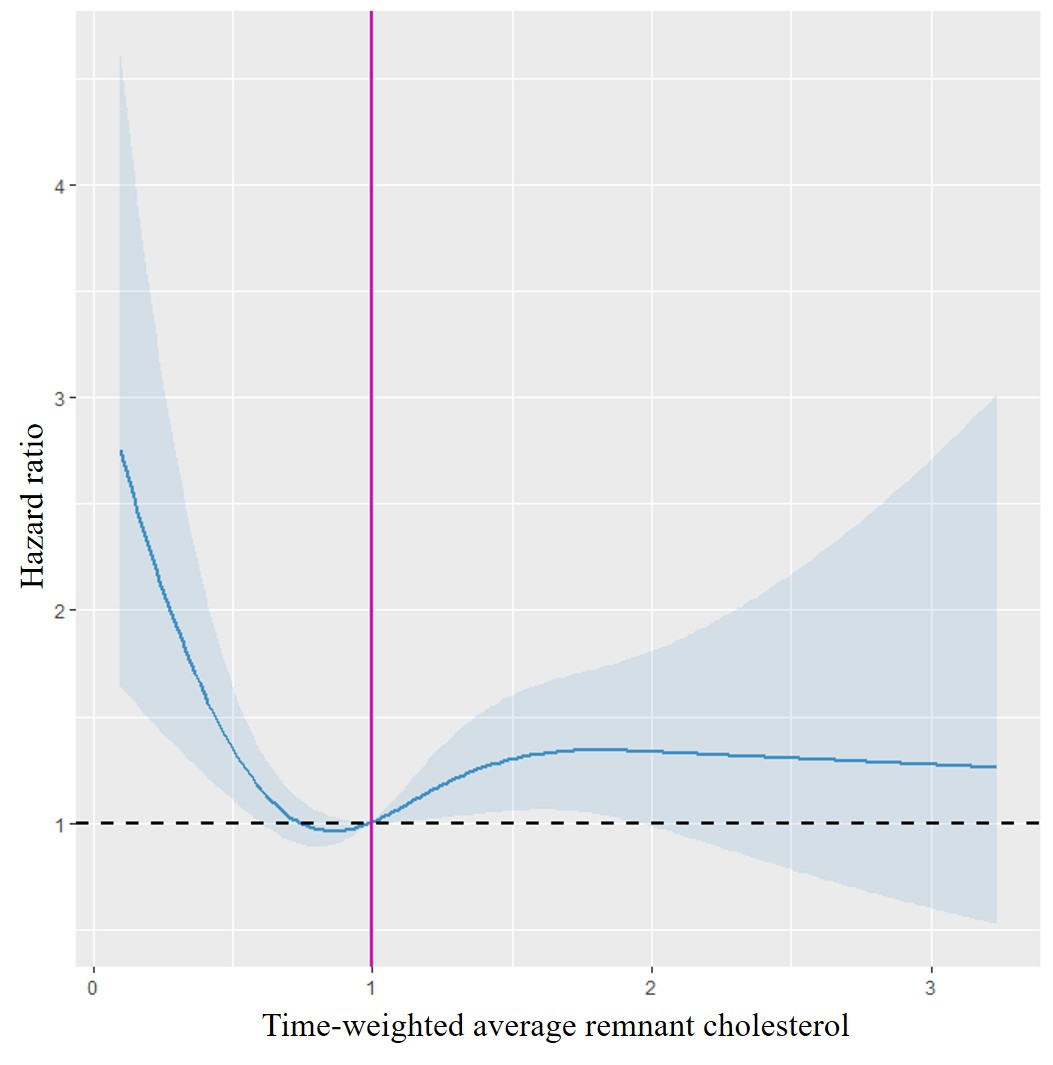

Supplement: Supplementary file 2 [file Image_1.tif]
